# Adapting and Developing A Diabetes Prevention Intervention Programme for South Africa: Curriculum and Tools

**DOI:** 10.3390/ijerph20054463

**Published:** 2023-03-02

**Authors:** Jillian Hill, Mieke Faber, Nasheeta Peer, Cindy George, Brian Oldenburg, Andre P. Kengne

**Affiliations:** 1 Non-Communicable Diseases Research Unit, South African Medical Research Council (SAMRC), Cape Town 7505, South Africa; 2 Center of Excellence for Nutrition, North-West University, Potchefstroom 2531, South Africa; 3 Department of Medicine, Faculty of Health Sciences, University of Cape Town, Cape Town 7700, South Africa; 4 Baker Heart and Diabetes Institute, School of Psychology and Public Health, La Trobe University, Victoria 3004, Australia

**Keywords:** diabetes prevention, intervention, healthy lifestyle curriculum, health education, South Africa

## Abstract

The South African Diabetes Prevention Programme (SA-DPP) is a lifestyle intervention targeting individuals at high risk of developing type 2 diabetes mellitus (T2DM). In this paper we describe the mixed-method staged approach that was used to develop and refine the SA-DPP intervention curriculum and the appropriate tools for local resource-poor communities. During the preparation phase, existing evidence on similar DPP interventions was reviewed, focus group discussions with individuals from the target population were conducted as part of a needs assessment, and experts were consulted. The curriculum booklet, a participant workbook and facilitator workbook were developed, and the content was evaluated by experts in the field. The design and layout of the booklet and workbooks needed to be culturally and contextually appropriate. The printed material was evaluated for readability and acceptability by participants of the target population; based on their feedback, the design and layout were refined and the printed material was translated. The suitability of the intervention was tested in a pilot study; based on feedback from the participants and facilitator, the curriculum was revised where needed and finalised. Through this process a context specific intervention and printed materials were developed. A complete evaluation of this culturally relevant model for T2DM prevention in South Africa is pending.

## 1. Introduction

It has been over two decades since the Finnish Diabetes Prevention Study (DPS) [1,2] and the Diabetes Prevention Program (DPP) in the United States [3] presented robust evidence on successful lifestyle intervention trials that reduced incident diabetes rates by 58%. Since then, several “real-world” (implemented in real-world conditions) diabetes prevention implementation trials and translational studies have been conducted, predominantly in high-income countries [4], including minority groups [5]. Only one (published) adaptation of a lifestyle intervention trial for diabetes prevention has been recorded in a low-income country, namely, India [6,7]. The original DPP included a high percentage of racial and ethnic minorities [3] and later adaptions thereof, for example, the YMCA model, included people of various ethnicities and socio-demographics [8]. However, a huge gap exists specifically in Africa.

The burden of type 2 diabetes (T2DM) in sub-Saharan Africa (SSA) is considerable and continues to rise rapidly. The number of people with diabetes in SSA is expected to increase from 24 million in 2021 to 55 million by 2045 [9]. In SSA, South Africa (SA) has the second largest number of people with diabetes. By 2017, there were 1,826,100 SA adults with diabetes [10], with the highest burden in socioeconomically disadvantaged populations in poorly resourced areas. A pooled analysis conducted as part of a systematic review and meta-analysis on the prevalence of diabetes in SA indicated that 11.3% (6.97–16.52 %) of the black population and 23.7% (13.93–15.73) of the mixed ancestry population have diabetes [11]. Global initiatives continually advocate for improved strategies in community-based approaches for T2DM prevention. Yet, little is known about such strategies in the African region.

The overall aim of the South African Diabetes Prevention Programme (SA-DPP) is to develop and evaluate a culturally relevant T2DM prevention approach for SA, based on the findings from successful diabetes prevention effectiveness and implementation programmes elsewhere [12,13,14,15]. The intention is to develop an exemplar that will inform lifestyle interventions aimed at preventing T2DM and other lifestyle-related conditions at the primary health care level in SA. This prototype may also serve as a model that can be adapted for other SSA countries facing similar challenges [16]. The SA-DPP lifestyle intervention proposes six group sessions based on empowerment ideology, emphasising the participant’s ability to make informed decisions, and his/her role as an independent decision maker who takes responsibility and regulates his/her own actions [17,18]. The group sessions are to be led by non-professional or community health workers (CHWs) (assisted by nurses and/or dieticians) and will be complemented by structured mobile phone messaging to augment adherence and retention. The lifestyle change objectives (diet and physical activity) of the SA-DPP are based on the original Finnish Diabetes Prevention Study, i.e., (1) < 30% of total energy intake from fat; (2) < 10% of total energy intake from saturated fat; (3) > 15 g of fibre/1000 kcal; (4) > 4 h/week moderate level of physical activity; and (5) > 5% reduction in body mass index [2]. In this paper we describe the process followed in developing an evidence-based and context specific lifestyle intervention curriculum and tools for the SA-DPP suited to lower socioeconomic communities, for adults from 25–65 years old at high risk of developing diabetes.

## 2. Methods and Process

**Figure 1:**
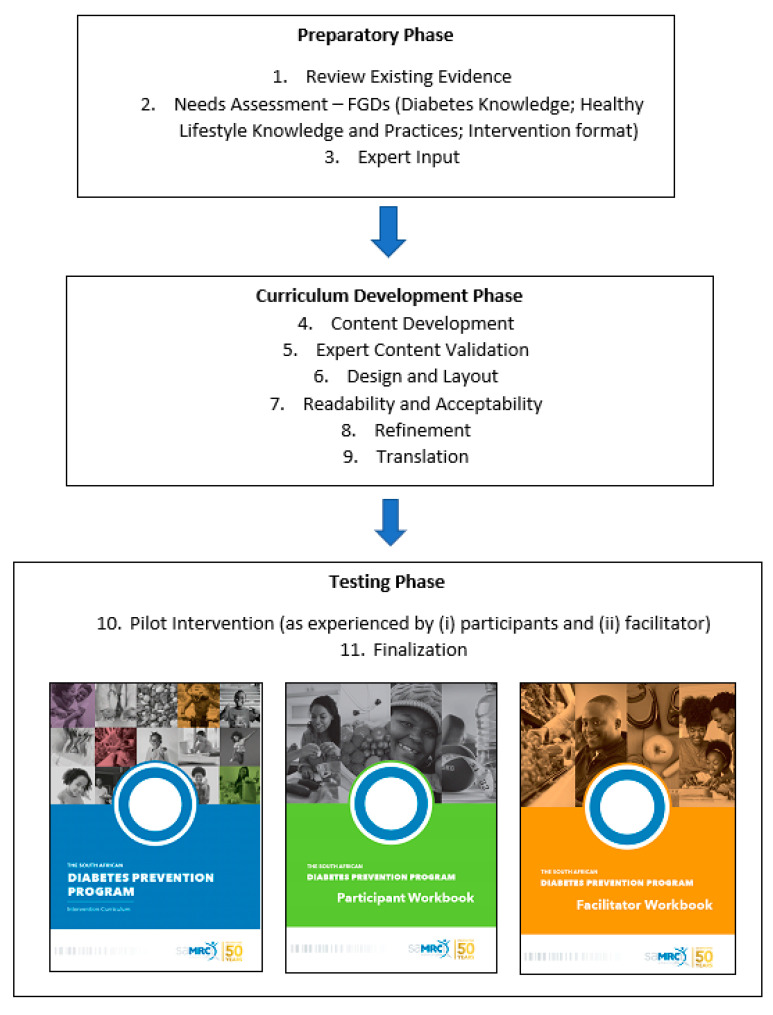
Process followed to develop the SA-DPP curriculum.

A qualitative mixed-method staged approach was followed in developing the SA-DPP intervention curriculum and tools (facilitator and participant workbooks). It included 11 steps, as follows: (1) reviewing and learning from existing evidence; (2) a needs assessment; (3) expert input; (4) development of the curriculum and tools; (5) expert content evaluation; (6) design and layout of the curriculum and tools; (7) participant readability and acceptability evaluation; (8) refining the design and layout of the curriculum and tools; (9) translating the curriculum and participant workbook; (10) suitability evaluation (pilot intervention); and (11) finalising the curriculum (see Figure 1). The research was approved by the Ethics Committee of the South African Medical Research Council (SAMRC) (approval no. EC018-7/2015).

### 2.1. Step 1: Reviewing and Learning from the Existing Evidence

Diabetes prevention implementation studies have gained prominence only over the past two decades. Implementation studies have been conducted primarily in Europe, North America, Australia, and more recently, India. These have been summarised from various perspectives in several systematic reviews over the last few years [19,20,21]. A consistent conclusion from these reviews is that there is sufficient evidence to support the reproducibility of the targeted lifestyle objectives and the efficacy of diabetes prevention trials in real-world settings, using less intensive interventions. However, the adaptation of DPPs to new settings has resulted in new programmes with different components, modes of delivery, duration, interventionists, target population, and outcomes [19,20]. This confirms that programmes must undertake the necessary processes for effective cultural adaptation in new settings. The review by Rawal et al., published in 2012, focused exclusively on developing countries and found only three efficacy or implementation trials on diabetes prevention, all conducted in India or China, with none in Africa [21]. Using the same search strategy as the latter review [21], we searched the literature published after 2012 to 2015 (in preparation for protocol submission) using PubMed/MEDLINE, and simplified and adapted the search terms to make them more specific to Africa by combining the following ‘diabetes’ AND ‘prevention’ AND [Africa OR “names for each African country”]; but found no ongoing or completed diabetes prevention study on the African continent. Subsequently, in preparation for intervention development we conducted a systematic review of the roles, responsibilities, and characteristics of the lay community health workers involved in diabetes prevention programmes [20], which rendered no papers from Africa. Evidence on the best strategies to implement diabetes prevention in Africa is therefore lacking.

The literature on adapted DPP interventions, similarly, is restricted to high-income countries and India [4,6,7,22]. Initial adaptions of the US DPP intervention included: (1) transforming the core curriculum from an intensive, individualised model to a group-based format; (2) removing costly toolbox incentives; (3) applying a formal exercise partner system (e.g., gym or sports club); and (4) delivering exercise components using local fitness club staff trained in behaviour change counselling rather than specialised life coaches [22]. The DPP lifestyle intervention was further adapted for the Group-Organized YMCA DPP (GO-YDPP), by merging the existing experiences and theory-driven adaptations of the intensive DPP lifestyle intervention to improve sustainability. The programme kept the physical activity and weight loss goals of the original DPP lifestyle intervention, but tailored these to meet individual needs with activities that were flexible, culturally sensitive, and acceptable to local communities. Emphasis was placed on self-esteem, empowerment, and social support. These principles were applied across the three key phases of their intervention, which comprised a core curriculum phase (16 lessons), a training and refinement phase (four weeks), and an ongoing maintenance phase [22].The Kerala Diabetes Prevention Program (K-DPP) was comprised of four key components as follows: (1) a group-based peer-support programme; (2) peer-leader training for lay people to lead groups; (3) resource materials (including a curriculum booklet and a participant workbook); and (4) strategies to inspire broader community engagement. The K-DPP adaptation employed a systematic approach, which was grounded in evidence-based behaviour change techniques [6].

### 2.2. Step 2: Needs Assessment

The needs assessment consisted of two rounds of focus group discussions (FGDs). Participants in the FGDs were recruited from amongst those who were part of the SA-DPP baseline screening pilot study that was conducted between August 2017 and March 2018 in eight resource-poor areas in Cape Town, Western Cape, South Africa [16]. The first four areas for which at least 20 individuals at risk of diabetes were identified were included in the needs assessment. Convenient sampling was employed whereby the first eight high-risk participants available per area were invited to participate in the FGDs. Two-to-three FGDs were conducted per area depending on the total number of participants. Two series of FGDs (17 in total) were conducted with 68 participants. Participants were mostly female (*n* = 50), between the ages of 45 and 65 years old (*n* = 58), and predominantly Xhosa speaking (*n* = 42) rather than English/Afrikaans speaking (*n* = 26) participants.

The aim of the first round of FGDs (*n* = 10) was to gauge participants’ knowledge and perceptions of diabetes and prevention, and to obtain inputs on the preferred intervention content, format, and delivery. The second round of FGDs (*n* = 7) focused specifically on lifestyle behaviours, i.e., diet and physical activity, and their related barriers and enablers. A focus group schedule was developed and used, which was informed using a combination of literature and consultation with SA-DPP project team members.

The FGDs were facilitated by a researcher (first author, trained in qualitative methodologies), while a second person (scribe) took notes. The participants were mainly English, Afrikaans, and Xhosa speakers. The FGDs were conducted predominantly in English as this was the language most suited to multiple language groups. The facilitator was fluent in both English and Afrikaans, and a Xhosa speaking scribe was present in the groups with Xhosa speaking participants. Participants were encouraged to respond in the language that they were most comfortable in. Thematic data analysis was employed to code and analyse the data, and have been described previously [23].

#### 2.2.1. Diabetes Knowledge and Perceptions

The themes and relevant quotes on participants’ knowledge and perceptions on diabetes are summarised in Table 1. From the FGDs it was clear that the participants understood that T2DM is a serious disease. Knowledge around the causes of T2DM was however limited, and participants mixed misperceptions with factual information. All participants knew someone with T2DM, who was often a family member, a friend, or a neighbour. Several participants were aware of T2DM complications and knew people with these complications (e.g., gangrene, leg amputation, slow healing of sores). In terms of diabetes prevention, participants were aware that lifestyle plays a role in diabetes occurrence. While they were not all certain that diabetes is preventable, they were keen to know more. A few felt that regular medical check-ups are key to preventing diabetes.

Participants had a fair idea about what healthy eating entails, including healthy food preparation. They knew that exercise is important and alluded to the importance of living a balanced lifestyle. When asked how confident they were in their ability to live a healthy lifestyle, participants in three of the 10 FGDs provided no comments, while the responses in the other FGDs were mixed. Most participants felt that household circumstances, i.e., family, and limited finances, would make it difficult to eat healthily and live a healthy lifestyle, while some felt confident that they could because it is for their own well-being. Factors that enabled self-efficacy were a positive mindset, cooking for themselves, and the ability to purchase healthy foods on a small budget. Participants identified various barriers to living a healthy lifestyle. Limited finances were the biggest challenge; this included having to feed a (large) family on a small budget. The availability of water and space for cooking was a challenge for individuals living in squatter camps (informal settlements). A lack of knowledge and family support, and laziness were also highlighted as barriers. Most participants had safety concerns when it came to physical activity outside the home. In the South African context, this is related to the high crime rates in the country, with outdoor activities generally being avoided and discouraged unless conducted in secure environments.

#### 2.2.2. Lifestyle Behaviours/Practices

The themes and relevant quotes on participants’ challenges, barriers, and enablers for healthy lifestyle behaviours are summarised in Table 2. Although some participants were attempting to live a healthy lifestyle, it seemed challenging for everyone. Again, the biggest barrier to eating healthy was financial constraints. The distance of the supermarkets from their home and knowledge on healthy eating were also mentioned. According to participants, factors that would enable them to eat healthier include greater financial spending power, their family’s buy-in and support, as well as bigger plots to plant their own vegetable gardens. Participants started to recognise the flaws in their perceptions on food and healthy eating and acknowledged that receiving the correct information and support would enable change. It was further evident that proper support from the community (for community vegetable gardens), the clinic (for useable information) and the ward councillor (for quality food parcels) is needed. The few participants who lived alone felt that eating healthy would be easy for them.

#### 2.2.3. Intervention Format

The themes and relevant quotes for participants’ perceptions on the proposed intervention format are summarised in Table 3. Participants were satisfied that group sessions held in the community would be the appropriate intervention format. There were some mixed responses regarding the facilitation of the group sessions; this did not necessarily have to be a CHW or peer leader. Some felt that it could be anyone, provided they are well trained and knowledgeable on the subject matter. Most participants were fine with English as the language of the intervention. However, for the intervention materials, while some preferred reading in English, others felt that these should be translated into their home language. Xhosa speaking participants felt more strongly about materials being in their home language than Afrikaans speaking participants.

During the second half of the first round of FGDs, participants were presented with an overall idea of what the intervention would entail, i.e., it would cover topics such as healthy eating and physical activity; it would be delivered via a group session; and that it was envisaged that sessions would be facilitated by a lay community health worker. The feedback received from participants, the themes and relevant quotes are summarised in Table 4. Participants requested that basic information on healthy eating should be included in the intervention curriculum. This should include topics such as a description of healthy foods and healthy eating guidelines, e.g., portions sizes, healthier meat options, etc. When asked whether they would have their family’s support to follow healthy eating guidelines, the responses were mixed with some families being willing to comply, while others refused to eat healthier food options. The latter makes it difficult for those who want to eat healthier, as financial constraints prevent the preparation of different meals for different household members. Although participants had a positive attitude towards physical activity and were familiar with the benefits thereof, their motivation to engage in such activities was lacking, with laziness being the key barrier. Other barriers included the community culture with exercise deemed unimportant, safety concerns, and a lack of facilities for physical activity. Participants felt that increased confidence would come with observing the benefits of exercise and learning about different exercises for specific body parts and injuries (e.g., knees and backache).

### 2.3. Step 3: Expert Input

The SA-DPP team were able to draw on the lessons learnt from the K-DPP, with access to the curriculum provided by a co-investigator who had collaborated on various DPP interventions, including the K-DPP. The K-DPP curriculum, developed for Kerala, a poor, small, densely populated state in south India, was strongly theory driven in its approach, drawing from behaviour change techniques [6]. A local co-investigator modified the curriculum, which was circulated to the SA-DPP multi-disciplinary team who were all non-communicable disease research scientists. The team included three dieticians, a physician, and a counsellor (psychologist). After the consultations, the original themes/topics covered in previous DPP curriculums (i.e., diabetes knowledge, healthy eating, physical activity, smoking, alcohol, and stress) and the behaviour change theories/techniques were included. However, these were adapted to be more relevant for the South African population, using the South African food-based dietary guidelines (SAFBDG) [24] as the basis.

The core project team concluded that the SA-DPP curriculum should be framed around the processes/stages of change as encapsulated by the transtheoretical model [25]. With the aim of moving the participants through the stages of contemplation, preparation, action, and finally the maintenance phase (with the expectation that once participants have been identified as at risk of developing diabetes at screening, they would be expected to be in the precontemplation/contemplation stage once they enter the DPP) [25]. Also, it was appreciated that in lifestyle programmes participants would likely never reach the termination stage, which indicates no temptation and 100% efficacy [25].

### 2.4. Step 4: Development of the Curriculum and Tools

A private practising dietitian was contracted to develop the SA-DPP curriculum and participant workbook, with guidance from one of the co-investigators, while the project manager developed the facilitator workbook.

*Curriculum Development:* Using the feedback from the FGDs, the topics were developed to provide basic knowledge together with practical tips to make lifestyle changes realistic and achievable. A section on setting SMART (specific, measurable, attainable, relevant, and timebound) goals was included. The curriculum content was kept as simple and relevant as possible for the target population considering their socioeconomic status (low income), educational level (matric or less), and environmental factors (safety and access).

*Participant Workbook:* The participant workbook was thereafter developed to encourage action-driven short- and long-term (realistic) goal attainment. The tasks in the workbook were goal oriented and subject specific (nutrition, physical activity, etc.), to enable participants to complete quick self-assessments and to set realistic goals, which they could then track with self-monitoring tools. The workbook also contained space for notes on self-reflections and in-session exercises.

*Facilitator Workbook:* The facilitator workbook, developed as a guideline/tool for facilitating the group sessions, included the roles and responsibilities of the facilitators, and a description and outline of each group session.

### 2.5. Step 5: Expert Content Evaluation

*Curriculum:* After finalisation, the curriculum was recirculated to the original expert group for confirmation that the content was valid and suitable for the study population.

*Participant and Facilitator Workbook:* The participant workbook and facilitator workbook were validated through an iterative process by two members of the multi-disciplinary team working independently and consultatively.

### 2.6. Step 6: Design and Layout of Curriculum and Tools

To ensure that the design and layout of the booklets, specifically the curriculum booklet and the participant workbook were culturally and contextually appropriate, the project manager worked closely with the South African Medical Research Council’s (SAMRC) Corporate and Marketing Division. The latter is the brand custodian of the SAMRC and is directed by the SAMRC’s broader organisational strategy and strategic goals. Important considerations for the design team were the target populations’ age, gender, ethnicity, and socioeconomic profile, including education level (for readability). Suitable photos and visuals were sourced and the layout, booklet size, letters, and spacing were mutually agreed upon. After a few rounds of engagement and amendments, the project team agreed that the format was acceptable.

### 2.7. Step 7: Participant Readability and Acceptability Evaluation

All participants who participated in the FGDs in the needs assessment phase were invited to participate in a workshop (conducted by members of the SA-DPP project team, with the first author as the lead facilitator) to test the readability and acceptability of the curriculum booklet and participant workbook. Only 16 participants were available to attend; four workshops (two Xhosa speaking and two Afrikaans/English speaking) were held with three to five participants each.

During these workshops, participants engaged with the printed materials, whereafter they completed a validated assessment tool [26] that was adapted for our setting and provided verbal feedback. The acceptability score was based on 38 questions pertaining to content (4), language (12), illustrations/photos (4), sufficiently specific/understandability of the information (6), legibility and printing characteristics (10), and the quality of information (2), using a 3-point Likert scale. An acceptability score was calculated as the sum of the scores for the 38 questions, expressed as a percentage. The mean acceptability score for the participant workbook was 94.3% (SD 7.6) and that for the curriculum booklet was 89.3% (SD 13.3).

From the verbal feedback received, participants felt that the booklets were very informative and easy to understand. Participants identified illustrations, figures, and photos that they were unhappy with, e.g., some photos showed vegetables not usually eaten in their communities, while others were of younger adults and children as opposed to people representing them, an older population. Most participants requested larger font sizes and a more simplistic front cover. Xhosa speaking participants expressed a strong need for the booklets to be translated into Xhosa.

### 2.8. Step 8: Refining the Design and Layout of the Curriculum and Tools

Based on the information provided by the participants (in Step 7), the design and layout of the curriculum and workbook were improved by redesigning the cover, increasing the font size, and reworking or replacing some illustrations and photographs.

### 2.9. Step 9: Translation of the Curriculum and Participant Workbook

The curriculum and participant workbook were translated into Xhosa by a language expert and verified by a native Xhosa speaker, via back translation. The translated version has the same design and layout as the English version.

### 2.10. Step 10: Suitability Evaluation (Pilot Study)

The overall aim of the pilot study was to test the suitability of the curriculum, tools, and group session format with a group of people at risk of T2DM. The planned intervention will comprise of bi-weekly sessions for the first five sessions with the sixth session at the end of month eight, with no plan for follow-up thereafter. The pilot study was an accelerated version of the latter with weekly sessions over a six week period (4–10 November 2020). The sessions were facilitated by a dietitian and co-facilitated by the first author onsite at the South African Medical Research Council (the COVID-19 pandemic precluded us from using community venues and recruiting a lay community health worker as a peer facilitator). The two smallest sites by the number of at-risk participants recruited (Du Noon (black) and Belhar (mixed ancestry)) were chosen as pilot sites. Out of the 20 participants (10 from each site) invited, only 10 (seven from Belhar and three from Du Noon) accepted the invitation to be part of the pilot study. The main reasons for declining the invitation related to the COVID-19 pandemic and their unavailability due to family commitments, i.e., taking care of grandchildren and other family members. The mean age of the participants in the pilot study was 54.6 (SD = 10.3) years.

#### 2.10.1. Summary of the Sessions as Experienced by Participants [Sessions 1–5: Table 5 and Table 6]

The process monitoring and evaluation, via evaluation sheets completed at the end of each session and verbal feedback, revealed great satisfaction with the intervention sessions. Session 2 (Healthy Eating Part 1) was the best attended (100%); all other sessions had about 70% attendance, collectively. Overall, most participants felt that they learnt something new in all the sessions, and exposure to information they already knew was still helpful. Across the sessions, an equal number of participants felt that the sessions were easy enough to follow vs. struggling at times. Participants found the self-assessments easy initially but needed some assistance as the sessions progressed. Participants experienced all the sessions as fun and engaging and looked forward to learning more. Most participants were excited and confident about making lifestyle changes. A few participants were anxious but determined to implement the lifestyle changes (Table 5).

**Table 5:** Intervention session log: session content.

Session:	1-Diabetes	2-Healthy Eating, Part 1	3-Healthy Eating, Part 2	4-Physical Activity	5-Smoking, Alcohol & Stress
No. of Participants	7	10	8	6	5
**How did you find today’s session?**					
I learned something new	**6**	**6**	**8**	**6**	**5**
Not all new, but the information was helpful	**3**	**5**	**1**	**1**	**2**
**I could follow the session easily**					
Yes, it was easy to follow	**7**	**5**	**4**	**5**	**3**
It was okay, but I struggled at times	**1**	**5**	**4**	**1**	**4**
**I found the self-assessment/goal setting to be…**					
Easy	**6**	**6**	**1**	**4**	**0**
Not too difficult but needed some help	**3**	**4**	**7**	**2**	**5**
**I found the session to be fun and engaging?**					
Yes, it was just right	**5**	**3**	**2**	**3**	**2**
I enjoyed it and look forward to the next session, I look forward to learning more	**7**	**10**	**8**	**6**	**5**
**I am excited to make some changes to my lifestyle**					
Yes, I am excited	**6**	**5**	**5**	**4**	**5**
I am excited, I know I can make lifestyle changes	**3**	**6**	**4**	**1**	**1**
I am anxious about it but determined	**3**	**1**	**2**	**2**	**1**
I am anxious and unsure whether I can do this	**1**	**0**	**0**	**0**	**0**

NB: In some instances, participants chose more than one answer.

The majority of participants indicated that the group sessions were sufficient to equip them with the knowledge and tools necessary to make lifestyle changes possible. Pertaining to the specific lifestyle changes adopted, most participants aimed to increase their fruit and vegetable intake, and physical activity levels, and maintain a healthy food plate. In rating the difficulty to introduce lifestyle changes, participants equally experienced it as easy, not too difficult but needing to figure out things, and challenging but determined to continue (Table 6).

**Table 6:** Intervention session log: goals and lifestyle changes.

Session:	1-Diabetes	2-Healthy Eating, Part 1	3-Healthy Eating, Part 2	4-Physical Activity	5-Smoking, Alcohol & Stress
No. of Participants	7	10	8	6	5
**What would make it possible to make lifestyle changes?**					
These group sessions will equip me with the knowledge and tools that I need	6	6	5	5	4
In addition to the group sessions, I will need the support of my family	1	4	3	0	0
**Tell us about the changes you started making…**					
Fruit & vegetables	*	2	2	3	5
Sugar intake	*	1	1	1	0
Fat intake	*	1	0	0	1
Healthy food plate	*	1	1	0	4
Physical activity	*	2	2	2	4
**How difficult is it to make changes to your lifestyle?**					
It has actually been easy!	**	**	3	3	2
It’s not been too difficult; however, I have a few things I must still figure out.	**	**	5	2	1
Even though it has been challenging I am determined to continue.	**	**	4	2	4

* Tell us about the changes you started to make, was an open-ended question in sessions 2, 3, and 4, and so all responses are not fully captured here and was not asked in session 1. ** Difficulty to make lifestyle changes not assessed in sessions 1–2.

#### 2.10.2. Session 6—Final Session: Check-in on Implemented Lifestyle Changes

Seven of the ten participants attended the final session, which consisted of qualitative feedback. The themes and relevant quotes are summarised in Table 7. All participants, irrespective of their perception of the interventions as easy or difficult, experienced the pilot study positively and received the information well. They applied the information not only to themselves, but also to their families, and the extended community, and became role models (and thus programme champions) for their community. Being able to share their knowledge with their friends, family, and community was a recurring theme throughout. Notably, one participant reported that her spouse suffered from uncontrolled diabetes and that by the end of the 6-week pilot study his diabetes was controlled, and he could move from insulin to oral medication only. The participant attributed this to her ability to apply the knowledge learnt in the group sessions to her household. The same participant’s son’s behaviour at school improved by simply replacing his juice intake with water. Those who smoked (*n* = 3) including the one person that consumed alcohol, reported struggling with cutting down on smoking and alcohol. However, they were aware of the need to reduce these behaviours and eventually plan to stop.

Participants found the curriculum and participant booklets extremely helpful, the curriculum booklet as a source of information and the participant workbook helped to keep them on track with their goals. Even though they found the goal setting (SMART goals) tough at first, they thought this was a good way to do it. Some of the participants did not keep track of their goals because they were busy or simply forgot. Participants found the intervention team to be easy going (non-judgmental) and supportive. Regarding the format of the programme, the content and the facilitators, the participants were satisfied and felt motivated. Their only suggestion was that friends and family could perhaps be included.

#### 2.10.3. Facilitator’s Reflections on the Sessions

The facilitator of the six weekly sessions of the pilot study was a bilingual (Xhosa and English speaking) dietitian, while the project manager took on the role of peer facilitator instead of the CHWs because of the COVID-19 restrictions. The facilitator noted that a few of the sessions ran over time, and that participants became more interactive and comfortable as the sessions progressed. The facilitator felt that the participants seemed to understand the information and enjoy the sessions and activities. The participants struggled slightly with the setting of SMART goals but were fine after some assistance. It was further noted that the use of the participant booklet for goal tracking at home was not well utilised. One of the sessions included an exercise on portion sizes, using food models. This highlighted two issues. Firstly, it demonstrated that participants grasped the concept of the divisions of the healthy food plate (1/2 a plate of non-starchy vegetables, ¼ starch, and ¼ protein), however portions were heaped on the plate. Secondly, the food models made the participants expectant and hungry, as it looked so real and life like.

### 2.11. Finalisation of the Curriculum

The curriculum was well received by all participants in the pilot study. The food plate exercise, however, revealed that a section with a direct focus on portion size was needed, which was subsequently added. Although the exercise with the food models was very useful, it created an expectancy that cannot be fulfilled by the research programme. Considering that SA-DPP is aimed at people in low-income communities, it was decided to remove the food models from the SA-DPP toolkit, for ethical reasons.

## 3. Discussion

The existing literature on the real-world adaptation of DPPs in high-income countries, including low-intensity DPPs, has shown varying effectiveness [4]. DPPs have also expanded to include adaptions suitable for different environments, such as the workplace [27,28], at schools targeting mothers, and via digital platforms targeting different audiences [29,30,31,32]. The importance of culturally tailoring interventions to specific minority groups and low socioeconomic populations has been recognised more and more over the years [5,33,34]. Interventions that are context specific and considered acceptable by the target population may have better adherence and therefore be more effective [35]. To our knowledge, a DPP adaption for low- and middle-income African countries has not yet been developed. Building on the evidence in the literature, the SA-DPP project team embarked on developing a context specific and culturally appropriate healthy lifestyle curriculum for South Africa, to ultimately be implemented in low socioeconomic populations at high risk of developing TSDM.

Although the objectives, principles, and themes of the Finnish Diabetes Prevention Study [1,12] and the K-DPP [6] form the core of the SA-DPP (Table S1), the SA-DPP curriculum is aligned with the nutrition messages from the South African Department of Health (i.e., the SAFBDG). The SAFBDG are short encouraging food-based dietary recommendations aimed at empowering the South African population to make healthier food choices (based on existing eating patterns) that will contribute to a nutritionally adequate diet and lower the risk of non-communicable diseases [24]. Behaviour change is, however, complex. The context specific barriers and opportunities for healthy eating should therefore be considered when adapting lifestyle interventions to promote population-level uptake without widening socioeconomic inequalities [36]. Barriers and enablers for healthy eating were explored during two rounds of FGDs in our study, and financial constraints was identified as the major barrier. It was, therefore, important to tailor the nutrition messages within the boundaries of the financial constraints of our target population, without compromising the intervention.

Sekhon et al. [35] suggest that the intervention needs to be acceptable to both the recipients and the facilitators of the intervention. When assessing acceptability prior to an intervention, the recipient’s perspective on the content, context, and perceived quality of the intervention is considered. Should an intervention be deemed acceptable, recipients are more likely to adhere to the intervention recommendations and benefit from it [35]. If the facilitators perceive the delivery to have low acceptability, the intervention may not be delivered as intended, and thereby impact its overall effectiveness [35]. Sekhon et al. [35] suggest a theoretical framework of acceptability with methodology for the development phase, the pilot or feasibility phase, the evaluation phase, and implementation phase.

A systematic review of culturally targeted strategies for diabetes prevention in minority populations by Lagissetty et al. [5], showed that, to be effective, interventions need to tailored across the following four domains: facilitators, language, location, and messaging. The SA-DPP intervention is a group based, peer support programme that is designed to educate and to provide support for people at high risk of developing T2DM. The groups will be jointly led by SA-DPP personnel and peer leaders selected from each community and trained on the SA-DPP intervention (**facilitators**/deliverers), who will deliver the intervention in the spoken/preferred language of the group (**language**). The purpose of the SA-DPP peer support groups (**messaging**/mode of delivery), which will take place in a community venue (**location**), is to provide a safe place where people who are at high risk of T2DM can meet regularly to support each other, develop friendships, share experiences, and learn from the experiences of others to improve their lifestyle. During the SA-DPP curriculum development process described in this paper, the intervention developers (experts/programme deliverers) and the intended recipients found the final SA-DPP curriculum and tools acceptable (including **language/readability/literacy competence and messaging,** i.e., the educational content), and the pilot study deemed it feasible. In essence giving the SA-DPP an increased chance of being effective.

The next phase of the SA-DPP, is the intervention phase (trial context) that will be implemented in 16 resource-poor communities. During the intervention phase, process evaluation techniques will be used to evaluate the curriculum, tools, and intervention on an ongoing basis. Process evaluation will be both formative and summative, to aid the dual purpose of helping fine tune the intervention during the implementation phase, and to evaluate the extent to which the intervention was implemented as planned and reached the intended participants. This information will in turn be used to assist the interpretation and description of the SA-DPP outcomes, analysing how the programme worked, and providing input for the future scaling up of the SA-DPP. Targeted elements of the implementation will include the fidelity, the dose (delivered and received), the reach, the recruitment, the retention/maintenance, and the context.

The biggest limitation to our study can also be viewed as its biggest strength in the context of tailoring an intervention to be culturally appropriate. Our intervention tools and curriculum have been developed with a specific focus on individuals at risk of developing T2DM in resource-poor communities in Cape Town, South Africa. Our process has enabled us to be mindful of key barriers and facilitators and has been demonstrated as was the case in Goff et al. [33], factoring in the importance of the context in which the intervention is aiming to effect change. However, our findings may not be generalisable to other contexts. Engaging the target population in the development of the intervention, the curriculum, and the tools, will promote ownership and inherent programme retention.

## 4. Conclusions

This paper presents the mixed-method staged approach that was followed in developing a context specific, culturally tailored intervention for diabetes prevention in South Africa, which can potentially be adapted for other African countries. Engaging the target population in the developing process ensured that individual and societal barriers and facilitators for healthy living are considered in the intervention and curriculum. Furthermore, engaging the target population promotes ownership and inherent programme retention. The intervention and curriculum were deemed acceptable, which would make intervention adherence and retention more plausible. Evidence in the literature strongly supports the potential effectiveness of culturally tailored interventions. The next step for the SA-DPP is a complete evaluation of this culturally relevant model for T2DM prevention in South Africa, which will provide some of the first evidence on the applicability and transferability of the findings from the international diabetes prevention efficacy trials in an African country.

## Figures and Tables

**Table 1:** Diabetes knowledge and perceptions: themes and quotes from the focus group discussions.

Themes	Supporting Quotations
Understood that diabetes is a serious disease	“I understand that diabetes is a very dangerous disease” (R5: FGD10). “A killer disease” (R6: FGD5).
Limited knowledge around the causes of diabetes	“Don’t know much [about] direct cause, [I] hear from people. If you eat too much fat, then you can get diabetes” (R2: FGD8).
Misperceptions mixed with factual information	“Can be passed on through the family. The way you eat can also cause diabetes, like eating fat” (R4: FGD3). “You can have diabetes even if you eat correctly. Sometimes it is inside your body” (R5: FGD3). “Used to say it’s a sickness for old people. Must take your tablets and eat healthily…” (R1: FGD2).
Complications of diabetes	“I know someone in my community whose legs were cut because of sugar diabetes” (R2: FGD2). “Whole family has diabetes. One sibling has gangrene. Sores take longer to heal with diabetes” (R1: FGD8). “A friend, did not take care of herself, did not eat properly and she became blind” (R1: FGD9).
Diabetes prevention	“It can be prevented, by eating properly…” (R2: FGD4). “Can’t prevent it completely. Should not stress, as that has an influence. Eating properly is very important…” (R5: FGD4). “Regular check-ups” (R2: FGD6). “Go to the clinics to get information, help about diabetes” (R1—FGD1). “I hope so. It depends on your lifestyle. If you want to be healthy. Exercise” (R6: FGD5).
Knowledge on healthy lifestyle	“Means less fatty meat, less sugar, less salt” (R2: FGD1). “Plant vegetables, limit oil and beef-stock intake and alcohol” (R4: FGD7). “Vegetables are important to eat. Do not have two starches” (R7: FGD8). “Drinking lots of water” (R3: FGD10). “Eat healthily. To prepare foods healthily. Not eating too much fruit and too much cereal. Hygiene related things. Exercise” (R1: FGD9). “Walk instead of taking public transport” R8: FGD2). “Exercise, keep moving. Do not eat too much. Keep a balance” (R2: FGD4).
Confidence to live a healthy lifestyle	“Not sure- finances…big families…lack of knowledge” (R4: FGD6). “I am not sure; I get tired easily and we buy cheap food” (R6: FGD6). “No. You will have a healthy home, but you eat bad elsewhere” (R2: FGD4). “If you want to live a healthy lifestyle and believe it then you can do it” (R2: FGD8). “Change begins with you- confident to start working on lifestyle” (R4: FGD1). “Confident, I do my own cooking” (R6: FGD1). “Yes, my mother was diabetic and it’s possible to eat healthily. Some things are cheaper than the unhealthy foods” (R5: FGD4). “…have already made means…small [vegetable] garden in the yard” (R2: FGD1).
Barriers to living a healthy lifestyle	“Money to an extent, especially if you have a family. Money is being shared. Some people do not have a choice. Water scarcity. Do not have knowledge of what to eat. Laziness” (R1: FGD9). “Living in a squatter camp with your children and cooking for everyone is a barrier. Money is a barrier, unable to buy healthier options such as brown sugar… No place to exercise” (R7: FGD3). “Knowing how to make certain foods [knowledge of food preparation]” (R5: FGD4). “Price and support from family, it is difficult to eat healthily when the family wants to eat unhealthily” (R2: FGD5). “Laziness is a barrier and not being determined to exercise” (R6: FGD3). “Places to exercise, it’s dangerous in the neighbourhood” (R6: FGD4).

**Table 2:** Lifestyle behaviours/practices; themes and quotes from the focus group discussions.

Themes	Supporting Quotes
Challenges to living a healthy lifestyle	“No, the food we eat is not healthy, eat a lot of fatty meats like intestines and braai meat and how we prepare food is not healthy if we cook cabbage, we fry it in oil. I just exercise and nothing more, I still eat fatty meat” (R2: FGD4). “To be healthy we were taught that you must cook food with oil and eat meat every day. But now we know that eating in this way is not necessarily right” (R4: FGD4).
Barriers to eating healthier	“We want to buy healthy food, but we don’t have the money to buy it” (R3: FGD4). “Shops like Shoprite are sometimes too far. So, distance is a barrier and sometimes the food at the community shops is not fresh and may even be rotten. The person who buys groceries does not have knowledge on food that is healthy, they buy fatty meat like polony and eggs” (R2: FGD4). “Another barrier is that the community is not supportive” (R2: FGD3).
Ability to eat healthier and possible enablers	“Cannot adopt it as I have to think of the family. I try to buy what I want that is healthier if there is money” (R2: FGD4). “I would need to relocate to another place that is big enough, so I can plant crops” (R2: FGD3). “The tradition of eating unhealthy foods from when you were small, is difficult to change” (R4: FGD1). “Change perception of ‘low fat’ tasting not as nice as ‘full cream’” (R1: FGD2). “Used to have a community garden but people started stealing” (R3: FGD1). “Clinics have papers informing you what to eat, but things listed are not always affordable. The [ward] councillor sometimes chooses two or three people or families who are poor and gives them food parcels but some of that food in the parcel is already rotten” (R2: FGD3). “It is not difficult if you live alone like I do. I can buy myself fruit and vegetables, but oil is difficult to reduce for me. So, I will try to eat healthy because I know now that the way I am eating is bad” (R1: FGD4).

**Table 3:** Participants’ perceptions on the proposed intervention format; themes and quotes from the focus group discussions.

Themes	Supporting Quotes
Facilitation of group sessions	“Someone from the outside community with knowledge and that won’t judge” (R6: FGD3). “I would prefer someone from outside our area, someone who does not know us, so that we can be free when we answer, someone who won’t judge (R8: FGD3). “The person should have knowledge, it doesn’t matter who it is” (R4: FGD3).
Language	“Yes, it is fine if it is in English. We, the group members can also assist each other with understanding the material” (R1: FGD10). “Both isiXhosa and English” (Group 2—R1: FGD6). “English is better for me” (Group 1—R1: FGD6). “The content should be in a language we understand” (R1: FGD1).

**Table 4:** Participants’ expectations, barriers, and enablers of a lifestyle intervention; themes and quotes from the focus group discussions.

Themes	Supporting Quotations
Healthy eating curriculum	“How to eat healthy, generally” (R5: FGD1). “What to and what not to buy” (R3: FGD4). “We want to know about food portions and what types of meat are healthy. More information on what is in the food and can advise community members” (R4: FGD4).
Healthy eating and familial support	“Yes, family eats what is cooked” (R1: FGD1). “Yes. Some of them will. Separate food will have to be made though” (R2: FGD1). “No. The family refuses to eat healthily” (R3: FGD1).
Knowledge on the benefits of physical activity	“When you exercise you feel light and relaxed” (R4: FGD4). “Exercise helps to prevent disease” (R3: FGD4). “Exercise makes your body healthy like if you have backache, you can do certain exercises to avoid having backache. It helps your mind-set as well, if you exercise you think faster, and exercise makes your body strong” (R5: FGD5).
Motivation to do physical activity	“There are no barriers we need to be disciplined; we are just lazy to exercise we sit the whole day” (R4: FGD5).
Physical activity practices	“I exercise daily I take a 5 km walk every day and even though I do have money to get a taxi sometimes I will walk” (R2: FGD6). “I am very fit. I wake up at 4 o’clock in the morning to do my work as a neighbourhood watch” (R3: FGD6).
Barriers to physical activity	“We don’t have gyms in the community. If we had gyms, we would exercise. At home there is not enough space so that limits us to exercise” (R1: FGD4). “Even though there is a park close to my community to exercise it is not safe. There are robbers waiting to rob you when you are done” (R2: FGD4). “Mostly I do it alone at home because there is no one around. I don’t want to go to my neighbour and say, ‘listen can we do exercises’, my neighbour will laugh at me, you know, because it is not something we do in the community” (R2: FGD2).
Physical activity curriculum	“What would increase my confidence is knowing what exercises to do because I have big arms, so I want to know what exercise I can do to them. My knees also lock sometimes so if you can teach us what exercising you can do for different parts of the body it would help” (R5: FGD5).

**Table 7:** Pilot study: participants’ overall experience; themes and quotes from the focus group discussions.

Themes	Supporting Quotations
Overall receipt of the intervention	“Last Sunday I wore a dress and my husband said I am glowing and I’m beautiful” (R1: FGD1). “No, you see for me, whenever I do something at home, whenever I get to it, then I’m thinking of these sessions because this makes part of my lifestyle… I was never drinking so much water… (R1: FGD2). “And my friends always ask me when they see me what happened today, they are getting interested” (R2: FGD2). “The kids are teasing ‘*You cannot drink cool drink*’ ‘MRC is waiting for you’, I say no that’s encouragement for me” (R3: FGD2). “Yes, yes, yes, no we pour it on the wife and kids, whenever my mom is there…” (R3: FGD2).
Community	“There is a difference in the community, I have shared information with my neighbours, things like giving advice on how to do things or portions to avoid high blood or diabetes and people are listening” (R2: FGD1). “One of them said, I’m not sure if it was yesterday or the day before yesterday, they were listening to the radio, and she said why are you telling us about things that Doctors from the radio say (laughter). I asked her what that was, she said that mum you tell us to drink water when we want things now the doctor says the same thing. I said it would be better if it is sinking in” (R1: FGD1).
Benefit to family	“It’s normal now, it’s not so high, and my husband’s sugar levels, we are healthy, I’m very happy” …[Husband] Sugar level was 46, [referring to doctor] “God has kept you because you were not supposed to have been able to wake up today”, he referred him to Somerset, he was dying… I mentioned before that I inject (husband); I stopped now. It’s alright now, every day they test him…” (R1: FGD1). “The principle asked me about this child; “[Participant’s name] what have you done to this child?” I asked him how do you mean? He said “Ay this child is relaxed now, he was naughty before” I will not defend him, he was really naughty… At school he said they must not give him juice, he wants water instead, what he sees at home he does at school” (R1: FGD1).
Struggle with smoking and alcohol	“I tried to smoke a little less but uhm I always come back again” (R1: FGD2). “The alcohol, you know I can stop at any time but when the friends come along there, they onto me again” (R1: FGD2). “I say it’s totally up to you yourself personally that you must just like setting a goal for yourself, but I am setting goals for myself now seriously. As I said my smoking is out of habit but my other goals that I have set for myself like losing a half a kilo because of me eating more fruit now and vegetables and I’ve reached that, I’ve lost a kilo now since I’ve started here” (R2: FGD2).
Curriculum and participant workbook	“It works very well, having these books works. It gives us knowledge; it helps us learn a lot” (R2: FGD1). “Even when you had forgotten something you could always go back to that page and read again. Even when you are talking to other people, for instance in my group I bring this book when I talk to people. I speak with them and refer to the book, it was like I was teaching them. They call me teacher when I get in the door” [R1: FGD1).
Difficulty with setting SMART goals	“At first it was difficult” (R2: FGD1). “Yes, but they are doable…I think this way is best” [R1: FGD1).
Slack in goal tracking	“I haven’t been writing, I’ve done all of the things and I’m thinking about you people, every time the wife is making the vegetables, I say oh okay you must start adding more colourings [colours] and there’s butternut, and there’s carrots and there’s squash and corns and all that” (R3: FGD2). “It’s not difficult to write down, it sometimes better to write down” (R3: FGD2).
Intervention team	“Another thing that keeps us interested is that if you experience any difficulty with the work at home when you get here you receive assistance…And not be scared to attend because we didn’t reach our goals and think no, I’m not going to class today because I didn’t do any work” (R1: FGD1). “You also as group leaders you make our lives also easy to follow you people, all your speeches, all your exercises, you make our lives easy with your program with that teaching” (R3: FGD2).
Format of intervention programme	“Just keep it. These sessions make a huge difference to me. It makes a difference to everybody because we eat differently, more healthier than before” (R3: FGD2). “Makes me feel better, it’s refreshing, keeps me motivated” (R4: FGD2). “Maybe we can bring friends along and have a bigger group” (R4: FGD2). “I think they are great the way they are now” (R2: FGD1).
Benefit to self	“All the things we learnt here come together, you can’t drink more water and still eat fat, you still eat an entire cake. I used to eat an entire cake, I won’t lie, I could eat an entire cake and still want another one after” (R1: FGD1). “In Xhosa we say “*Isiqhelo siyayoyisa ingqondo*” (habit overpowers the brain) and it really does because you are doing what you are used to, and the brain gets overpowered” (R2: FGD1). “What I benefit [ed] from this program is that I’m more conscious of how I’m eating now, I share it with my friends who also started changing some of their eating habits, have reached some goals using less sugar, lost one kilo, still have some goals to achieve like trying to smoke less or stop completely. I benefit a lot from this program, my lifestyle is better, it’s improved because I’m eating better with more fruit, less sweetie stuff, my only habit is the cigarette habit”. [R3: FGD2]

## Data Availability

Not applicable.

## Supplementary Materials

The following supporting information can be downloaded at: https://www.mdpi.com/article/10.3390/ijerph20054463/s1, Table S1: Intervention Components of Finnish, Australian and Indian DPP.
